# Expectations, Body Image Pressure, and Willingness to Undergo Cosmetic Procedures Among First-Time Aesthetic Plastic Surgery Seekers: A Cross-Sectional Study

**DOI:** 10.7759/cureus.115835

**Published:** 2026-09-05

**Authors:** Asma Maryam, Zurnish Rauf, Sidra Arif, Usman Nazir

**Affiliations:** 1 Medicine, Shifa International Hospital Limited, Islamabad, PAK; 2 Surgery, University of Health Sciences, Lahore, PAK; 3 Surgery, Mohtarma Benazir Bhutto Shaheed Medical College, Mirpur, PAK

**Keywords:** aesthetic surgery, body image pressure, cosmetic procedures, patient expectations, plastic surgery, social media, willingness

## Abstract

Background

Interest in aesthetic surgical and non-surgical procedures has increased alongside greater public visibility of cosmetic treatment and widespread exposure to appearance-focused social media content. First-time aesthetic procedure seekers may approach consultation with varying levels of body image pressure, social influence, and expectations regarding appearance, confidence, and social acceptance.

Objective

This study aimed to assess expectations, body image pressure, and willingness to undergo cosmetic procedures among first-time aesthetic plastic surgery seekers and to identify factors associated with willingness to proceed with treatment.

Methods

A cross-sectional study was conducted among 200 adults attending a plastic surgery and aesthetic medicine clinic for their first aesthetic consultation. Participants with no previous history of cosmetic surgical or non-surgical treatment were recruited through consecutive sampling. Data were collected using a structured, study-specific questionnaire covering sociodemographic characteristics, preferred aesthetic procedures, treatment expectations, body image pressure, appearance-related social media exposure, and willingness to undergo a cosmetic procedure. Categorical variables were summarized using frequencies and percentages, while age was reported as mean and standard deviation. Associations were assessed using the chi-square test, and binary logistic regression was performed to identify factors independently associated with willingness. Adjusted odds ratios were reported with 95% confidence intervals, and p < 0.05 was considered statistically significant.

Results

The mean age of participants was 28.6 ± 7.4 years, and 132 (66.0%) were female. High body image pressure was identified in 124 participants (62.0%), while 138 (69.0%) reported willingness to undergo a cosmetic procedure. Willingness differed significantly across body image pressure categories (χ²(2) = 27.29, p < 0.001; Cramér’s V = 0.37). In multivariable analysis, high body image pressure showed the strongest association with willingness (adjusted odds ratio (AOR): 3.84; 95% confidence interval (CI): 1.92-7.68; p < 0.001). Frequent appearance-related social media exposure (AOR: 2.41; 95% CI: 1.28-4.54; p = 0.006), expectation of improved self-confidence (AOR: 2.76; 95% CI: 1.39-5.48; p = 0.004), and previous negative comments about appearance (AOR: 1.93; 95% CI: 1.01-3.69; p = 0.046) were also significantly associated with willingness. Sex and age group were not statistically significant after adjustment.

Conclusion

Among first-time aesthetic procedure seekers, body image pressure, frequent appearance-related social media exposure, expectation of improved self-confidence, and previous negative comments about appearance were associated with greater willingness to undergo cosmetic procedures. These findings support careful pre-procedure counselling and assessment of appearance-related concerns, expectations, and social influences. Because of the cross-sectional design, causal relationships cannot be established. Multicentre and longitudinal studies using validated psychological measures are needed to clarify the temporal and contextual factors influencing cosmetic-procedure decisions.

## Introduction

Aesthetic plastic surgery and other cosmetic procedures have become increasingly common worldwide, with substantial growth in both surgical and non-surgical interventions. International data reported approximately 34.9 million aesthetic procedures in 2023, reflecting the continued expansion of cosmetic treatment services [1,2]. This growth has occurred alongside advances in surgical techniques, wider availability of minimally invasive procedures, and greater public visibility of aesthetic treatment. However, the reasons individuals consider cosmetic procedures are complex and cannot be explained by procedural availability alone.

Treatment-seeking behaviour may be influenced by a combination of psychological, social, and cultural factors. Body image concerns, perceived social judgement, comparison with others, and expectations of improved confidence may all contribute to interest in aesthetic treatment [3]. Body image reflects an individual’s perceptions, thoughts, and feelings regarding physical appearance, and persistent dissatisfaction with a facial or body feature has been associated with greater acceptance of cosmetic procedures [4]. These relationships are important because appearance-related concerns may interact with broader social experiences rather than operate as isolated influences.

Social media may further shape the context in which appearance concerns develop. Platforms such as Instagram, TikTok, Facebook, and Snapchat expose users to idealized and digitally altered images, beauty filters, influencer content, and frequent appearance-based comparisons [5]. Previous studies have linked repeated exposure to such content with body dissatisfaction, social appearance anxiety, and greater consideration of cosmetic procedures [6,7]. However, these associations do not establish that social media directly causes treatment-seeking behaviour, and individuals already interested in cosmetic procedures may also be more likely to engage with appearance-focused online content.

Patient expectations represent another important component of aesthetic decision-making. Some individuals seek correction of a specific feature or improvement in facial or body proportion, whereas others may anticipate broader psychosocial benefits, including greater self-confidence, social acceptance, improved relationships, or professional advantage [8]. Although many patients report satisfaction after cosmetic treatment, less favourable psychosocial outcomes may occur when expectations are unrealistic or when significant appearance-related distress is not identified before treatment [9]. For this reason, understanding both the physical and psychosocial expectations of first-time seekers is clinically relevant.

First-time aesthetic procedure seekers are particularly important because they may have limited previous experience of procedural risks, recovery, possible complications, and the limitations of cosmetic outcomes [10]. They may also be more vulnerable to unrealistic comparisons with edited photographs, filtered images, or idealized online content. Body dysmorphic disorder and severe appearance-related distress have been reported more frequently among cosmetic treatment seekers than in the general population [11,12]. The present study did not diagnose body dysmorphic disorder, but assessment of appearance-related concerns and expectations remains relevant to safe patient selection and counselling.

The cultural and social context in which cosmetic procedures are considered may also influence treatment-seeking behaviour. Such contextual factors may differ across populations and clinical settings and may interact with individual body image concerns, expectations, and social influences. However, evidence examining these factors jointly among first-time aesthetic procedure seekers in Pakistan remains limited. Existing research has more commonly examined body dissatisfaction, social media exposure, patient expectations, and attitudes toward cosmetic procedures as separate factors, while evidence regarding their combined association with willingness to proceed with treatment remains limited.

Therefore, this study aimed to assess expectations, body image pressure, and willingness to undergo cosmetic procedures among first-time aesthetic plastic surgery seekers and to identify factors associated with willingness to proceed with treatment. By examining these factors together in a Pakistani clinical setting, the study sought to provide context-specific evidence that may support more informed pre-procedure counselling and patient assessment.

## Materials and methods

This cross-sectional study was conducted among first-time aesthetic procedure seekers attending the outpatient department of a tertiary-care plastic surgery and aesthetic medicine clinic in Islamabad, Pakistan, from January 2025 to June 2025. The study assessed patient expectations, body image pressure, appearance-related social media exposure, and willingness to undergo cosmetic surgical or non-surgical procedures. The manuscript was prepared in accordance with the Strengthening the Reporting of Observational Studies in Epidemiology (STROBE) recommendations for cross-sectional studies [13].

First-time aesthetic procedure seekers were defined as individuals attending their first-ever consultation for a cosmetic surgical or non-surgical procedure and having no previous history of cosmetic surgical or non-surgical treatment. The inclusion and exclusion criteria used for participant selection are summarized in Table 1.

**Table 1:** Inclusion and Exclusion Criteria

Inclusion criteria	Exclusion criteria
Adults aged 18 years or older	Seeking reconstructive treatment for trauma
Attending their first-ever aesthetic consultation	Seeking reconstructive treatment for burns
No previous history of cosmetic surgical or non-surgical treatment	Congenital abnormalities requiring treatment
Provided written informed consent	Functional impairment or another medically indicated condition
—	Unable to understand or complete the questionnaire
—	Declined written informed consent
—	Questionnaire with more than 20% missing responses

The sample size was calculated using the single-population proportion formula. In the absence of a reliable local estimate of willingness to undergo cosmetic procedures, an expected proportion of 50% was used. At a 95% confidence level and 7% absolute precision, the minimum required sample size was 196. The target was increased to 200 participants to account for incomplete questionnaires. Eligible participants were recruited consecutively until the required sample was achieved.

Data were collected using a structured questionnaire developed after reviewing relevant literature on body image, cosmetic procedure acceptance, patient expectations, and social media influence. The questionnaire collected sociodemographic information, preferred aesthetic procedure, treatment expectations, body image pressure, appearance-related social media exposure, previous negative comments about appearance, and willingness to undergo a cosmetic procedure. The questionnaire was reviewed by two plastic surgeons, one dermatologist, and one clinical psychologist for relevance, clarity, and appropriateness of the items and was pilot-tested among 20 first-time aesthetic consultation attendees who were not included in the final sample. Minor revisions were made for clarity and sequence. The six body image pressure items demonstrated acceptable internal consistency (Cronbach’s alpha = 0.81). No formal psychometric validation against an established external instrument was performed; therefore, the questionnaire was treated as a study-specific assessment tool rather than a validated diagnostic instrument. The complete study questionnaire is provided in Appendix A, the variable coding and scoring scheme is presented in Appendix B, and a summary of questionnaire development and validation is provided in Appendix C.

Body image pressure was assessed using six yes-or-no items addressing dissatisfaction with a facial or body feature, comparison with others, social media-related appearance pressure, negative comments from family or peers, distress when viewing photographs or selfies, and avoidance of social situations because of appearance concerns. Each affirmative response received one point, producing a total score of 0-6. Scores of 0-1, 2-3, and 4-6 were categorized as low, moderate, and high body image pressure, respectively. These categories were used as study-specific operational groupings for descriptive and analytical purposes and were not intended to represent established clinical or diagnostic thresholds.

Participants could select multiple expectations from cosmetic treatment, including improved appearance, greater self-confidence, better social acceptance or professional image, improved relationship prospects, correction of a disliked feature, and a desire to appear younger. Appearance-related social media exposure was classified as frequent when participants reported viewing relevant content daily or on at least five days per week and as infrequent when exposure occurred on fewer than five days per week.

Willingness to undergo a cosmetic procedure was recorded as willing, unsure, or not willing. For binary logistic regression, ‘willing’ was coded as 1, while ‘unsure’ and ‘not willing’ were combined and coded as 0. This grouping was used to distinguish participants who expressed a definite willingness to proceed from those who had not made a definite positive decision at the time of consultation. Written informed consent was obtained before questionnaire administration. Participants completed the questionnaire privately, and personal identifiers were excluded from the analytical dataset. Complete-case analysis was performed without statistical imputation.

Data were analysed using IBM SPSS Statistics (IBM Corp., Armonk, NY, USA) for Windows, version 26.0. Age was summarized as mean and standard deviation, while categorical variables were reported as frequencies and percentages. Associations between categorical variables were assessed using the chi-square test, with Fisher’s exact test used when expected cell frequencies were insufficient. Binary logistic regression was performed to identify factors independently associated with willingness to undergo a cosmetic procedure. The final model included body image pressure, appearance-related social media exposure, expectation of improved self-confidence, sex, age group, and previous negative comments about appearance. Sex was recorded as male or female in the study questionnaire; gender identity was not assessed separately.

For regression analysis, low and moderate body image pressure were combined as the reference category and compared with high body image pressure. Social media exposure was categorized as frequent versus infrequent, sex as female versus male, and age as 18-30 years versus above 30 years. The age categories were used as a pragmatic analytical grouping within the study sample and were not intended to represent clinically defined age thresholds. Adjusted odds ratios with 95% confidence intervals were reported. Multicollinearity was assessed using tolerance and variance inflation factors, model calibration using the Hosmer-Lemeshow goodness-of-fit test, and explanatory performance using Nagelkerke R². All tests were two-sided, with p < 0.05 considered statistically significant.

Temporary ethical approval was obtained from the Institutional Review Board and Ethics Committee of Shifa International Hospitals Limited, Islamabad, Pakistan (IRB #229-24) on December 10, 2024, before the commencement of data collection. Subsequent approval documentation for the same study was issued on July 12, 2026.

## Results

During the study period, 218 individuals were assessed for eligibility. Eight individuals did not meet the inclusion criteria, four declined participation, and six returned questionnaires with more than 20% missing responses. A total of 200 first-time aesthetic plastic surgery seekers were included in the analysis. The mean age of the participants was 28.6 ± 7.4 years. The detailed sociodemographic characteristics of the participants are presented in Table 2.

**Table 2:** Sociodemographic Characteristics of the Study Participants Most participants were young adults, with the largest proportion aged 26-35 years. Females represented 66.0% of the study population, and nearly half of the participants were graduates.

Variable	Category	Frequency (n)	Percentage (%)
Age group	18-25 years	74	37.0
	26-35 years	86	43.0
	36-45 years	28	14.0
	Above 45 years	12	6.0
Sex	Female	132	66.0
	Male	68	34.0
Marital status	Unmarried	112	56.0
	Married	84	42.0
	Divorced/widowed	4	2.0
Education	Up to intermediate	38	19.0
	Graduate	96	48.0
	Postgraduate	66	33.0
Employment status	Student	54	27.0
	Employed	92	46.0
	Self-employed	26	13.0
	Unemployed/household work	28	14.0

The distribution of primary aesthetic procedures sought by participants is presented in Table 3.

**Table 3:** Preferred Aesthetic Procedures Among First-Time Aesthetic Plastic Surgery Seekers Each participant selected one primary aesthetic procedure. Rhinoplasty was the most commonly preferred procedure, followed by liposuction/body contouring and botulinum toxin or fillers. Surgical and non-surgical aesthetic procedures were both represented in the study population.

Preferred procedure	Frequency (n)	Percentage (%)
Rhinoplasty	50	25.0
Liposuction/body contouring	38	19.0
Botulinum toxin/fillers	34	17.0
Skin-related aesthetic procedures	30	15.0
Hair transplant	18	9.0
Breast procedures	16	8.0
Facial rejuvenation surgery	14	7.0

Improvement in physical appearance and self-confidence were the most commonly reported expectations from cosmetic treatment. Expectations related to social acceptance, professional image, relationships, correction of a disliked feature, and younger appearance are summarized in Table 4.

**Table 4:** Expectations From Cosmetic Procedures Multiple responses were permitted; therefore, percentages do not total 100%. Improved physical appearance and increased self-confidence were the most frequently reported expectations. Correction of a long-standing disliked feature was also commonly reported.

Expectation	Frequency (n)	Percentage (%)
Improved physical appearance	158	79.0
Improved self-confidence	144	72.0
Better social acceptance	104	52.0
Improved professional image	78	39.0
Better marriage/relationship prospects	62	31.0
Correction of a long-standing disliked feature	118	59.0
Desire to look younger	54	27.0

Dissatisfaction with a specific facial or body feature was the most frequently reported appearance-related concern. Comparison with others, social media pressure, and distress related to photographs or selfies were also common. The individual body image pressure indicators are presented in Table 5.

**Table 5:** Body Image Pressure Indicators Among Participants Participants could report more than one indicator; therefore, percentages do not total 100%. Dissatisfaction with a specific facial or body feature was the most common body image pressure indicator. Frequent comparison with others and social media-related appearance pressure were also prevalent.

Body image pressure indicator	Frequency (n)	Percentage (%)
Dissatisfaction with specific facial/body feature	152	76.0
Frequent comparison with others	128	64.0
Social media-related appearance pressure	122	61.0
Negative comments from peers/family	74	37.0
Distress in photographs/selfies	116	58.0
Avoidance of social situations due to appearance	52	26.0

Using the study-specific operational categories defined in the Materials and Methods, most participants were classified as having high body image pressure. The distribution across low, moderate, and high categories is shown in Table 6.

**Table 6:** Overall Level of Body Image Pressure Most participants were classified as having high body image pressure, while a smaller proportion had low body image pressure.

Level of body image pressure	Frequency (n)	Percentage (%)
Low	26	13.0
Moderate	50	25.0
High	124	62.0

Detailed willingness responses are presented in Table 7.

**Table 7:** Willingness to Undergo Cosmetic Procedures

Willingness status	Frequency (n)	Percentage (%)
Willing	138	69.0
Unsure	42	21.0
Not willing	20	10.0

Willingness to undergo a cosmetic procedure increased across the body image pressure categories and was highest among participants with high body image pressure. The association between body image pressure and willingness to undergo a cosmetic procedure was statistically significant, χ²(2) = 27.29, p < 0.001, with a Cramér’s V of 0.37, indicating a moderate effect size, as shown in Table 8.

**Table 8:** Association Between Body Image Pressure and Willingness to Undergo Cosmetic Procedures df, degrees of freedom; n, number of participants. The chi-square statistic, degrees of freedom, Cramér’s V, and p-value refer to the overall association across body image pressure categories.

Body image pressure	Willing, n (%)	Unsure/not willing, n (%)	χ²	df	Cramér’s V	p-value
Low	11 (42.3)	15 (57.7)	27.29	2	0.37	<0.001
Moderate	25 (50.0)	25 (50.0)				
High	102 (82.3)	22 (17.7)				

In the multivariable analysis, high body image pressure showed the strongest adjusted association with willingness to undergo a cosmetic procedure. Frequent social media exposure, expectation of improved self-confidence, and previous negative comments about appearance were also significantly associated with willingness. Sex and age group were not statistically significant after adjustment. Full regression estimates are presented in Table 9.

**Table 9:** Multivariable Logistic Regression Analysis of Factors Associated With Willingness to Undergo Cosmetic Procedures AOR, adjusted odds ratio; CI, confidence interval. The dependent variable was willingness to undergo a cosmetic procedure, coded as willing versus unsure/not willing. Reference categories were low/moderate body image pressure, infrequent social media exposure, no expectation of improved self-confidence, male sex, age above 30 years, and no previous negative comments about appearance. The logistic regression model demonstrated acceptable calibration according to the Hosmer-Lemeshow goodness-of-fit test (χ² = 6.24, p = 0.621). The model explained 31.4% of the variation in willingness to undergo cosmetic procedures according to Nagelkerke R² and correctly classified 77.5% of participants. No substantial multicollinearity was identified, as all variance inflation factor values were below 2.0.

Predictor	Adjusted odds ratio (AOR)	95% Confidence interval	p-value
High body image pressure vs low/moderate	3.84	1.92-7.68	<0.001
Frequent vs infrequent social media exposure	2.41	1.28-4.54	0.006
Expected improved self-confidence vs did not expect	2.76	1.39-5.48	0.004
Female vs male	1.62	0.84-3.12	0.148
Age 18-30 years vs above 30 years	1.71	0.89-3.29	0.106
Prior negative comments about appearance vs none	1.93	1.01-3.69	0.046

## Discussion

This cross-sectional study examined expectations, body image pressure, appearance-related social media exposure, and willingness to undergo cosmetic procedures among first-time aesthetic plastic surgery seekers. More than two-thirds of participants reported willingness to proceed with a cosmetic procedure. In the adjusted analysis, high body image pressure showed the strongest association with willingness, while frequent appearance-related social media exposure, expectation of improved self-confidence, and previous negative comments about appearance were also significantly associated with willingness [14,15]. Female sex and younger age showed higher odds of willingness, but these associations were not statistically significant. Overall, the findings suggest that willingness among first-time aesthetic procedure seekers may be related to a combination of appearance-related concerns, social influences, and expectations of psychosocial benefit. Although several associations were statistically significant, their clinical relevance should be interpreted in the context of individual patient assessment rather than as direct predictors of treatment decisions. The findings may be most useful in identifying areas for discussion during pre-procedure counselling.

High body image pressure was common and was strongly associated with willingness to undergo a cosmetic procedure. Dissatisfaction with a specific facial or body feature, comparison with others, distress related to photographs or selfies, and perceived social media-related appearance pressure were frequently reported [16,17]. This pattern is consistent with previous literature showing that dissatisfaction with appearance is associated with greater acceptance of cosmetic procedures and interest in appearance modification. However, body image pressure in the present study was assessed using a study-specific scoring system rather than a validated psychiatric diagnostic instrument [18]. Therefore, these findings should be interpreted as evidence of appearance-related pressure rather than evidence of body dysmorphic disorder. Taken together, the findings suggest that appearance-related concerns, social comparison, and expectations of psychosocial benefit may operate as interconnected influences on willingness, although the cross-sectional design does not allow the direction of these relationships to be established.

Frequent exposure to appearance-related social media content was independently associated with willingness to undergo cosmetic procedures. Visual social media platforms may increase opportunities for appearance comparison and repeated exposure to idealized, edited, or digitally enhanced images [19]. However, the present study cannot determine whether social media exposure influenced treatment-seeking decisions or whether individuals already interested in cosmetic procedures were more likely to engage with appearance-focused content. Importantly, the questionnaire did not directly assess whether changing beauty ideals or reduced social stigma toward cosmetic procedures motivated participants to seek treatment. These factors should therefore be considered contextual explanations rather than findings established by the present study. The observed association nevertheless supports considering appearance-related social media use during pre-procedure assessment [20].

Expectation of improved self-confidence was another factor significantly associated with willingness. Many participants expected cosmetic treatment to improve physical appearance and confidence, while some also anticipated greater social acceptance, professional benefit, or improved relationship prospects [21]. These expectations are clinically relevant because cosmetic procedures may improve satisfaction with a specific physical feature but may not necessarily resolve broader psychological distress, low self-esteem, social anxiety, or interpersonal concerns. Pre-procedure counselling should therefore distinguish realistic, procedure-specific goals from expectations of major changes in confidence, social status, relationships, or professional success.

Previous negative comments about appearance were also associated with greater willingness to undergo cosmetic procedures. This finding suggests that external social experiences, including criticism or teasing from family members or peers, may be relevant to treatment-seeking behaviour. However, the timing, frequency, source, and emotional impact of such comments were not assessed, and the nature of this relationship could therefore not be examined in detail. Future studies should evaluate whether repeated appearance-related criticism contributes independently to body dissatisfaction and procedural interest [22].

Female sex and younger age were associated with higher odds of willingness, but these associations were not statistically significant after adjustment. These findings should be interpreted cautiously and should not be taken to indicate that demographic characteristics are unimportant. Willingness to undergo cosmetic procedures may also be shaped by cultural expectations, socioeconomic circumstances, family and peer influences, and other contextual factors that were not fully captured in the present study [11,23]. The single-centre setting in Islamabad may further limit the extent to which the findings represent other cultural, socioeconomic, or regional groups in Pakistan.

The findings have practical implications for aesthetic practice. First-time seekers may benefit from structured consultation that evaluates motivations, expectations, body image concerns, appearance-related social media exposure, and previous appearance-related criticism [23]. Patients with high appearance-related pressure or broad psychosocial expectations may require more detailed counselling before a treatment decision is made. Where severe distress, persistent preoccupation, marked functional impairment, or clinically unrealistic expectations are identified, referral for psychological assessment may be appropriate. Such an approach may support informed consent, realistic expectation setting, and safer patient selection [5,24].

Limitations

This study has several limitations. Its cross-sectional design prevents conclusions about causality or the temporal direction of the observed associations. The use of consecutive sampling in a single clinical setting may limit generalizability to other hospitals, regions, cultural groups, and socioeconomic populations. The findings were based on self-reported responses and may therefore be affected by recall and social desirability bias. Body image pressure and appearance-related social media exposure were assessed using study-specific measures rather than fully validated psychometric instruments, which may limit comparability with other studies. Gender identity was not assessed separately, and detailed socioeconomic, ethnic, and cultural variables were not collected. The questionnaire also did not include an open-ended question exploring participants’ reasons for considering cosmetic enhancement, which may have limited the ability to capture motivations beyond the predefined response options. In addition, combining “unsure” and “not willing” into one category for binary regression may have reduced potentially meaningful differences between these groups. The regression model explained 31.4% of the variation in willingness, indicating that other unmeasured psychosocial, cultural, socioeconomic, and contextual factors may also contribute to treatment decisions. Future multicentre and longitudinal studies using validated instruments, broader demographic measures, and qualitative or open-ended approaches are needed to better define these relationships and establish their temporal direction.

## Conclusions

Among first-time aesthetic procedure seekers, high body image pressure, frequent appearance-related social media exposure, expectation of improved self-confidence, and previous negative comments about appearance were associated with greater willingness to undergo cosmetic procedures. These findings highlight the value of assessing appearance-related concerns, expectations, and social influences during pre-procedure counselling, while recognizing that the cross-sectional design does not establish causality. This study contributes evidence from a Pakistani clinical setting and supports the need for multicentre and longitudinal research using validated psychological measures to better clarify the temporal and contextual factors influencing cosmetic-procedure decisions.

## Appendices

Appendix A 

**Table 10:** Study Questionnaire The body image pressure items were study-specific operational measures. Detailed scoring and analytical coding are provided in Appendix B.

Section	Item	Question / Response Options
A. Sociodemographic Characteristics	1	Age: ______ years
	2	Sex: Male / Female
	3	Marital status: Unmarried / Married / Divorced or Widowed
	4	Education level: Up to Intermediate / Graduate / Postgraduate
	5	Employment status: Student / Employed / Self-employed / Unemployed or Household Work
B. Preferred Aesthetic Procedure	6	Which primary aesthetic procedure are you seeking consultation for? Rhinoplasty / Liposuction or body contouring / Botulinum toxin or fillers / Skin-related aesthetic procedure / Hair transplant / Breast procedure / Facial rejuvenation surgery
C. Expectations From Cosmetic Treatment	7	What do you expect from the cosmetic procedure? Multiple responses permitted: Improved physical appearance / Increased self-confidence / Better social acceptance / Improved professional image / Better marriage or relationship prospects / Correction of a long-standing disliked feature / Desire to appear younger
D. Body Image Pressure	8	Are you dissatisfied with a specific facial or body feature? Yes / No
	9	Do you frequently compare your appearance with that of others? Yes / No
	10	Do you feel appearance-related pressure from social media? Yes / No
	11	Have you received negative comments about your appearance from family members or peers? Yes / No
	12	Do you experience distress when viewing photographs or selfies of yourself? Yes / No
	13	Do you avoid social situations because of concerns about your appearance? Yes / No
E. Appearance-Related Social Media Exposure	14	How frequently do you view appearance-focused social media content, beauty filters, digitally altered photographs, influencer posts, or information related to cosmetic procedures? Daily / At least five days per week / Fewer than five days per week
F. Willingness to Undergo a Cosmetic Procedure	15	How would you describe your willingness to undergo a cosmetic procedure? Willing / Unsure / Not willing

Appendix B

**Table 11:** Variable Coding and Scoring Scheme Body image pressure categories were study-specific operational classifications and were not intended to represent validated clinical or diagnostic thresholds.

Variable	Measurement / Response	Coding or Classification
Body image pressure items	Six Yes/No items	Yes = 1; No = 0
Total body image pressure score	Sum of six items	Possible score: 0-6
Low body image pressure	Total score 0-1	Low category
Moderate body image pressure	Total score 2-3	Moderate category
High body image pressure	Total score 4-6	High category
Body image pressure in regression	Low / Moderate / High	Low + Moderate = reference group; High = exposure group
Appearance-related social media exposure	Daily / at least five days per week / fewer than five days per week	Frequent = daily or ≥5 days/week; Infrequent = <5 days/week
Willingness to undergo a cosmetic procedure	Willing / Unsure / Not willing	Three categories used for descriptive analysis
Willingness in binary regression	Willing / Unsure / Not willing	Willing = 1; Unsure + Not willing = 0
Expectation of improved self-confidence	Yes / No	Binary variable
Previous negative comments about appearance	Yes / No	Binary variable
Sex in regression	Female / Male	Female compared with Male as reference
Age group in regression	18-30 years / Above 30 years	18-30 years compared with >30 years as reference

Appendix C

**Table 12:** Questionnaire Development and Validation Summary Cronbach’s alpha, measure of internal consistency.

Component	Description
Questionnaire development	The structured questionnaire was developed after reviewing relevant literature on body image, acceptance of cosmetic procedures, patient expectations, and social media influence.
Main domains assessed	Sociodemographic characteristics, preferred aesthetic procedure, treatment expectations, body image pressure, appearance-related social media exposure, previous negative comments about appearance, and willingness to undergo a cosmetic procedure.
Expert review	The initial questionnaire was reviewed by two plastic surgeons, one dermatologist, and one clinical psychologist.
Purpose of expert review	To assess the relevance, clarity, and appropriateness of the questionnaire items.
Pilot testing	The questionnaire was pilot-tested among 20 first-time aesthetic consultation attendees.
Inclusion of pilot participants	Participants involved in pilot testing were not included in the final study sample.
Changes following pilot testing	Minor revisions were made to improve wording, clarity, and question sequence.
Body image pressure assessment	Six Yes/No items were used to assess appearance-related pressure.
Internal consistency	The six body image pressure items demonstrated acceptable internal consistency, with a Cronbach’s alpha of 0.81.
Nature of body image measure	The body image pressure items and score categories were developed specifically for this study and were used as operational measures rather than validated diagnostic criteria.
Diagnostic purpose	The questionnaire was not designed to diagnose body dysmorphic disorder or any other psychiatric condition.
